# OsLIC, a Novel CCCH-Type Zinc Finger Protein with Transcription Activation, Mediates Rice Architecture via Brassinosteroids Signaling

**DOI:** 10.1371/journal.pone.0003521

**Published:** 2008-10-27

**Authors:** Lei Wang, Yunyuan Xu, Cui Zhang, Qibin Ma, Se-Hwan Joo, Seong-Ki Kim, Zhihong Xu, Kang Chong

**Affiliations:** 1 Key Laboratory of Photosynthesis and Environmental Molecular Physiology, Institute of Botany, the Chinese Academy of Sciences, and National Centre for Plant Gene Research, Beijing, China; 2 Department of Life Science, Chung-Ang University, Seoul, Korea; Massachusetts General Hospital, United States of America

## Abstract

Rice architecture is an important agronomic trait and a major limiting factor for its high productivity. Here we describe a novel CCCH-type zinc finger gene, *OsLIC* (*Oraza sativa* leaf and tiller angle increased controller), which is involved in the regulation of rice plant architecture. *OsLIC* encoded an ancestral and unique CCCH type zinc finge protein. It has many orthologous in other organisms, ranging from yeast to humane. Suppression of endogenous *OsLIC* expression resulted in drastically increased leaf and tiller angles, shortened shoot height, and consequently reduced grain production in rice. *OsLIC* is predominantly expressed in rice collar and tiller bud. Genetic analysis suggested that *OsLIC* is epistatic to *d2-1*, whereas *d61-1* is epistatic to *OsLIC*. Interestingly, sterols were significantly higher in level in transgenic shoots than in the wild type. Genome-wide expression analysis indicated that brassinosteroids (BRs) signal transduction was activated in transgenic lines. Moreover, transcription of *OsLIC* was induced by 24-epibrassinolide. OsLIC, with a single CCCH motif, displayed binding activity to double-stranded DNA and single-stranded polyrA, polyrU and polyrG but not polyrC. It contains a novel conserved EELR domain among eukaryotes and displays transcriptional activation activity in yeast. OsLIC may be a transcription activator to control rice plant architecture.

## Introduction

Rice (*Oryza sativa*) is one of the most important crops and a model plant for monocots. Rice yield is mainly modulated by its architecture [1]–[3], which is defined by tillering number and angle, internodes elongation, panicle morphology and leaf angle [4], [5]. Selection of certain plant architecture is critical for dense planting and for improving lodging resistance during rice cultivation. The *sd1* gene, encoding OsGA20ox2 [6], [7], also termed the “Green Revolution” gene, confers semi-dwarf stature and significantly contributes to increased rice production. MOC1 (MONOCULM 1), one of the GRAS family members, plays an important role in controlling tillering. The *moc1* mutant plants have only one main culm without any tillers because of the defect in the formation of tiller buds [1]. Recently, tiller angle was reported to be controlled by a major quantitative trait locus, *TAC1* (Tiller Angle Control 1), which was mapped to a 35-kb region on chromosome 9 [8]. Leaf angle also is an important agronomic traits in rice varieties [3]. New rice cultivars with erect leaves, which increases light harvest for photosynthesis and grain filling, may have increased grain yield [2]. In the other hand, leaf angle is a significant morphological marker for the brassinosteroids (BR) response in rice [9]. Blocking either BR biosynthesis or its signal transduction pathway in rice results in erect leaves. In contrast, rice seedlings treated with BRs show increased leaf angle in a dose-dependent manner [10]–[13].

CCCH-type zinc finger proteins belong to an unusual zinc finger protein family containing tandem zinc-binding motifs characterized by three cysteines followed by one histidine (CX_7–8_CX_5_CX_3_H; X represents any amino acid) [14]. A typical CCCH protein usually contains two tandem CCCH-type zinc-binding motifs separated by 18 amino acids [14]. Such proteins are present widely in eukaryotes, from yeast to mammals. Through their zinc fingers, these proteins can bind to mRNAs containing class II AU-rich elements (AREs), generally at their 3′-untranslated regions (3′-UTR). Tristetraprolin (TTP), also known as TIS11, NUP475 and GOS24) is an example of this family in mammals [14]–[16]. TTP inhibits TNF-alpha production from macrophages by destabilizing its mRNA through directly binding to the ARE of the TNF-alpha mRNA [17]. PIE-1, POS-1, MEX-1 and MEX-6 are the other CCCH-type zinc finger proteins, with two copies of CCCH zinc finger motifs, that specify the identity of germline blastomeres in early embryonic development in *C. elegans* [18]–[21]. These results demonstrate that CCCH-type zinc finger proteins are key developmental regulators in *C. elegans* that specify the fates of early embryonic cells.

In plants, HUA1, a CCCH-type zinc finger protein with 6 tandem CCCH motifs, is able to associate with *AGAMOUS* mRNA to regulate its mature process to indirectly determine organ identity specification [22]. Recently, another CCCH-type zinc finger, *FRIGIDA-ESSENTIAL 1 (FES1)*, was found to be required for the up-regulation of *FLC* expression and for the FRI-mediated winter-annual habit [23]. Besides binding to mRNA and influencing its metabolism, CCCH-type zinc proteins also regulate gene expression in distinctive mechanisms. For example, the human CCCH-type zinc finger protein TTP/TIS11/NUP475 may be involved in activating transcription [24]. PIE-1 is also required for efficient expression of the maternally encoded *Nanos* homolog *NOS-2* at the post-transcriptional level in *C. elegans* [19]. Thus, CCCH-type zinc finger proteins can regulate gene expression from the transcriptional to posttranscriptional level. However, less is known about how CCCH-type zinc finger proteins function as transcriptional regulators in higher plants.

Here, we show that *OsLIC* (Oraza sativa leaf and tiller angle increased controller) is critical in regulating rice plant architecture. Down-regulation of *OsLIC* by an antisense approach in rice conferred multiple architecture-related phenotypes, including increased leaf angle, tiller angle, and reduced plant height. Our results suggest that OsLIC functions as a negative regulator for optimal plant architecture in rice through mediating the BR response, probably via acting as a negative regulator in sterol homeostasis. Moreover, a novel conserved EELR domain in OsLIC appears to be functional as a transcriptional activator.

## Results

### Phenotypes of *OsLIC* antisense transgenic plants

**Figure 1:**
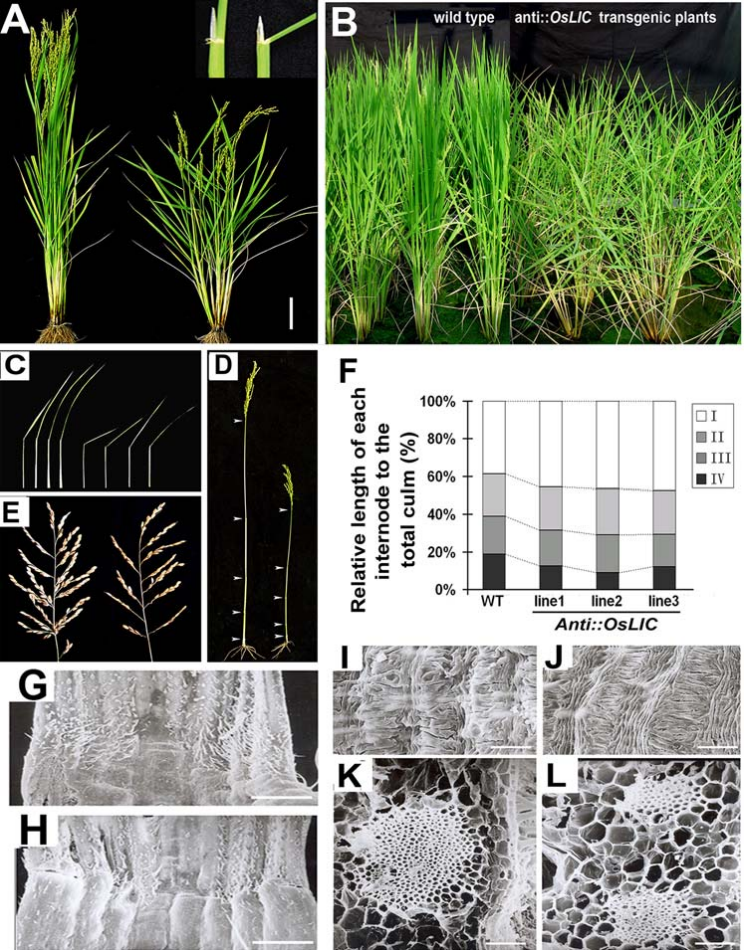
Phenotypes of antisense *OsLIC* transgenic plants. (A) Antisense *OsLIC* transgenic plants displayed multiple phenotypes, such as increased leaf angle, semi-dwarfism and increased tillering in mature period. Close-up view of lamina joint of wild-type plants and that of transgenic plants is shown in the right top corner of Figure 1A to display significantly increased leaf angle in transgenic plants. Bars = 10 cm. (B) T3 progeny of antisense *OsLIC* transgenic plants displayed similar phenotypes as T2 progeny in the field. (C) Comparison between morphology of four upper leaves in wild-type and transgenic plants. (D) Height of the upper four nodes; arrowhead indicates internodes. (E) Panicle morphology of transgenic plants showing reduced number of spikelets and seeds. From A–E, wild type plants are placed in the left part, and *AntiOsLIC* transgenic plants are placed in the right part. (F) Analysis of plant height of transgenic plants. From up, the first, second, third and fourth nodes respectively. (G to J) Cell morphology and arrangement of collar abaxial surface in transgenic plants significantly differed from that of wild-type plants. In addition, vascular bundles were markedly smaller in cross-sections on scanning electron microscopy (K and L). Bars = 1 mm in G and H; Bars = 100 µM in I and J. Bars = 20 µM in K and L.

To screen genes controlling architecture in rice, we used a reverse genetics approach to study the functions of transcript-factor genes using a microarray containing 10K cDNAs [25]. An EST named y722d04p5 corresponding to a putative CCCH-type zinc finger protein (AK107008) was identified on the basis of preponderant expression in the stem node [26]. This gene was designated as *Oryza sativa* leaf and tiller angle Increased Controller (*OsLIC*) based on its phenotypes. DNA gel blot analysis and BLASTx search results revealed the gene is a unique gene in rice genome (data not shown), which allows for investigating its biological function with a reverse genetics approach. Thus, an antisense full-length cDNA of *OsLIC* under the control of the maize *ubiquitin1* promoter (*Ubi: AntiOsLIC*) was transformed into rice to investigate biological function. After *Agrobacterium*-mediated transformation, 14 independent hygromycin-resistant T_0_ plants were regenerated from hygromycin-resistance callus and transferred to soil. Harvested T1 seeds were grown on hygromycin-supplemented media to screen for single-copy integration of T-DNA according to their separation ratio. Three positive T_1_ lines containing single T-DNA insertion sites were obtained and further proved by DNA gel blot analysis following hygromycin resistance screening (Figure S1A). The expression of OsLIC protein was suppressed in these antisense lines (Figure S1B). The transgenic plants of *AntiOsLIC* conferred multiple phenotypes in tillering and heading stages. Leaf and tiller angles were greatly increased in transgenic plants as compared to wild-type plants (Figure 1A, B, C and D, Table 1). The transgenic plants were also shorter than the wild type (Figure 1D). Deduction percentage of the length of different internodes differed in the transgenic plants from that in the wild type (Figure 1F). Moreover, transgenic lines displayed both reduced number of rachises and number of seeds in a panicle (Figure 1E and Table 1). Among the altered phenotypes observed, increased leaf angle was one of the most dramatic defective phenotypes. Scanning electronic microscopy (SEM) of the transgenic plants at heading stage showed more parallel protuberances in the adaxial surface of collars of transgenic plants than those of the wild type (Figure 1 G and H). Cell arrangement also was greatly altered in collars of transgenic rice as compared with the wild type (Figure 1I and J). In addition, cross-sections of collars revealed markedly smaller vascular bundles in *AntiOsLIC* lines than in the wild type (Figure 1K–L).

**Table 1:** Phenotypes of *OsLIC* transgenic rice plant lines.

	Wild type	Line 1	Line 2	Line 3
Height (cm)	83.2±5.32	68.9±6.39	73.6±4.4	71.4±4.35
Leaf angle	19.8±8.74	66.58±5.07	77.67±7.15	44.4±8.0
Number of rachis in each panicle	14.11±1.48	10.15±1.37	9.68±1.56	10.71±1.61
Number of seeds in each panicle	113.92±13.07	67.07±16.78	67.35±12.78	69.18±16.41

Note: mean±SD from 15 individual plants.

### Genetic analysis of *OsLIC* transgenic plants with rice BRs mutants

**Figure 2:**
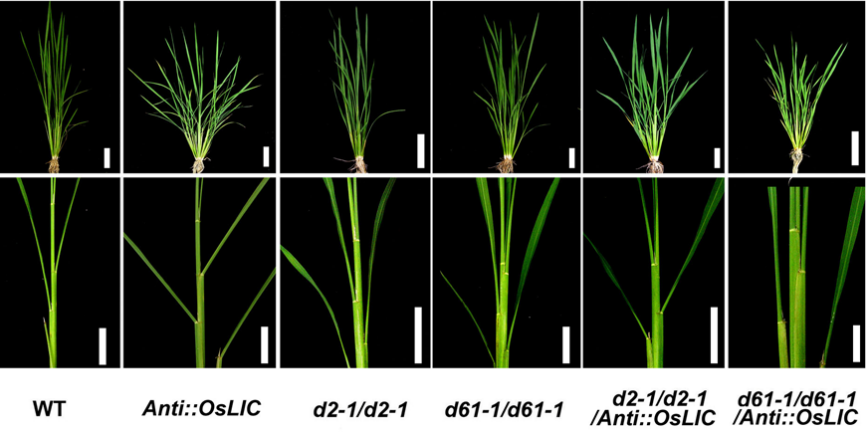
Epistatic analysis of antisense *OsLIC* transgenic plants. Antisense OsLIC transgene (male parent) was crossed with *d2-1* and *d61-1* mutants plants (female parents). The phenotypes of representative plants containing *AntiOsLIC* transgene in homozygous *d2-1/d2-1* and *d61-d61* background were shown in tillering period among F2 progeny. Bars = 10 cm.

Rice BRs mutants such as *d2-1* with mutation in *D2/CYP90D2* and *d61-1* with mutation in *OsBRI* show erect leaves [10], [12], which is opposite to the phenotype of *AntiOsLIC* transgenic lines. To examine the genetic relationship between *OsLIC* and BR biosynthesis or signaling mutants, we crossed the antisense lines (as a male parent) with the homozygous mutants *d2-1* and *d61-1* (as a female parent) respectively [10], [12]. Similar to the antisense lines, all F1 progenies showed drastically increased leaf angle, which indicated that cross was successful (data not shown). Then, we screened the homozygous *d2-1* and *d61-1* mutation by sequencing the F2 progenies as was reported previously [10], [12]. F2 plants containing both *Ubi::AntiOsLIC* and homozygous *d2-1* showed increased leaf angle similar to *Ubi::AntiOsLIC* lines (Figure 2), whereas F2 plants containing both *Ubi::AntiOsLIC* and homozygous *d61-1* showed erect leaves (Figure 2). These results suggest that *OsLIC* is epistatic to *d2-1*, while *d61-1* is epistatic to *OsLIC*.

### Expression pattern of *OsLIC* in rice

**Figure 3:**
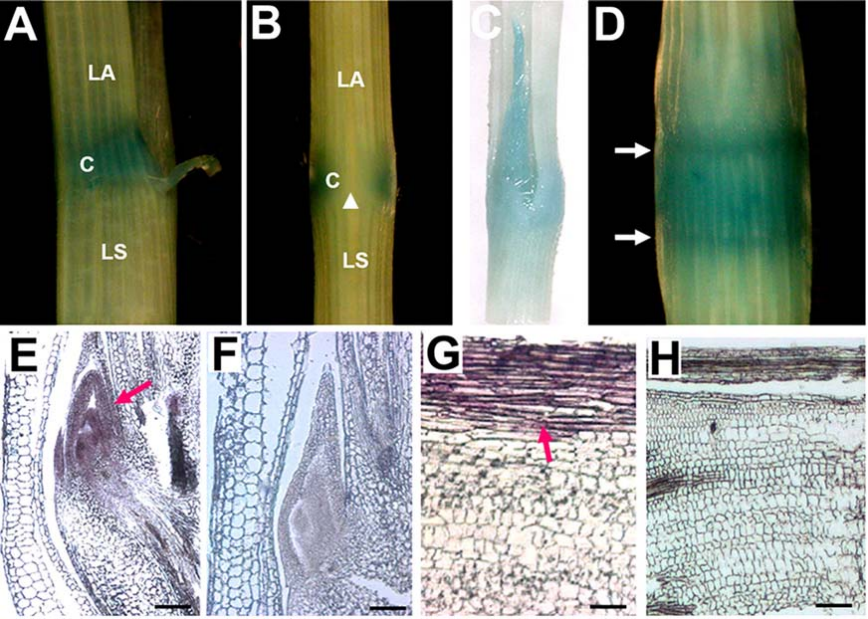
Expression pattern of *OsLIC*. (A–D) The native promoter (−2234 to −1 bp) of *OsLIC* drives the *UidA* gene expression to investigate its expression pattern in the T2 generation. Signals are predominately detected in the collar (C), and a weak signal was detected in lamina (LA) and leaf sheath (LS) (a, c). GUS activity is not observed in the adaxial surface of the collar, as shown by the arrowhead (B). GUS signal is also strongly detected in nodes (D). (E) *In situ* hybridization demonstrated *OsLIC* predominantly expressed in the collar and (G) in the outgrowth stage of tillering primodia; (F and H) sense probe was used as negative control.

To test whether the reduced expression of *OsLIC* caused the defective phenotypes in transgenic plants, we transformed the marker gene ß-glucuronidase (GUS) driven by the *OsLIC* native promoter (2.3 kb) into rice plants. As shown in Figure 3A, higher GUS activity was detected in the collar, which is consistent with the phenotype of increased leaf angle in transgenic plants. In addition, we also detected strong signals in nodes and the basal region of elongating internodes (Figure 3B–D), where cell division and elongation are active at tillering and heading stages. In contrast, lamina and leaf sheaths showed low levels of GUS (Figure 3A). Interestingly, a strong signal was detected only in the adaxial surface but not the abaxial surface of the collar (Figure 3B). High GUS activity was detected in young collars, which were even not sprouted from the last leaf sheath (Figure 3C). The predominant expression of *OsLIC* mRNA in the collar and basal regions of nodes is consistent with the defective phenotypes observed in transgenic plants (Figure 1A and 1H). Consistently, *in situ* hybridization showed strong *OsLIC* signal in adaxial cells and tillering primodia (Figure 3E and 3G) but no signals with the sense probe used as a control (Figure 3F and 3H).

### Sterol profiles were altered in transgenic plants

**Figure 4:**
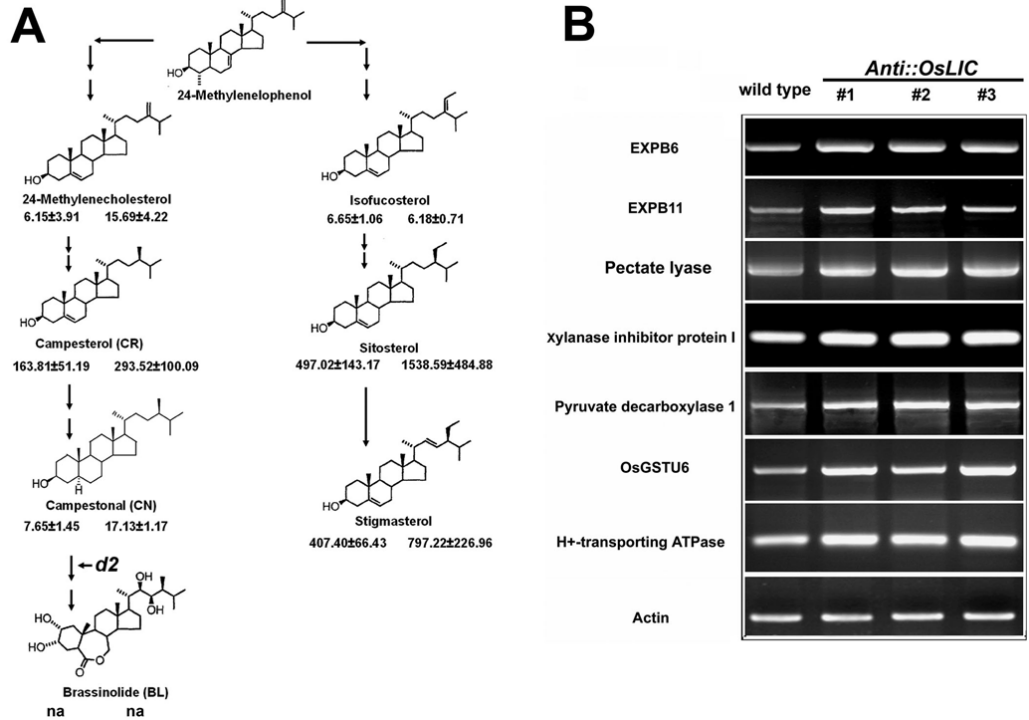
Sterol profile assay in *OsLIC* transgenic plants. (A) Increased sterol abundance in transgenic plants (right panel) than in wild-type plants (left panel). Data are means±SE from three trials. (B) RT-PCR analysis confirmed the increased expression of genes associated with cell-wall assembly in transgenic plants, which have been identified by microarray analysis.

Sterols are a group of molecules that are structurally similar to BRs. Previous studies indicated that sterols, including typical sterols (sitosterol and stigmasterol) and atypical sterols (8, 14-diene sterols accumulated in *fk* mutants), affect the expression of genes involved in cell expansion and cell division [27]. In addition, brassinolide, sitosterol, stigmasterol, and the atypical *fk* sterol CH can all induce the expression of *TCH4*, *Meri*-*5*, β-*tubulin*, and *KOR* in Arabidopsis [27]. BR biosynthesis and signal transduction are conserved in both rice and Arabidopsis [28]–[32]. Since *OsLIC* is epistatic to *d2-1* and *d61-1* is epistatic to *OsLIC*, OsLIC is probably involved in the regulation of sterol abundance to indirectly affect BRs response in rice. To test this hypothesis, we examined the sterol profile in *AntiOsLIC* lines and wild type plants. As shown in Figure 4A, the level of sitosol and stigmasterol in *AntiOsLIC* shoots was 3.05- and 1.95-fold higher than in wild-type shoots respectively, whereas that of 24-methylenechosterol (a common precursor of stigmasterol, campesterol and brassinolide) and isofucosterol was similar in both *AntiOsLIC* and wild-type plants (Figure 4A). The sterol abundance profiles were not significantly changed in roots (data not shown). Thus, OsLIC likely functions as a fine-tuning regulator of sterol homeostasis in rice.

### Genome-wide gene expression profile analysis of transgenic plants

**Figure 5:**
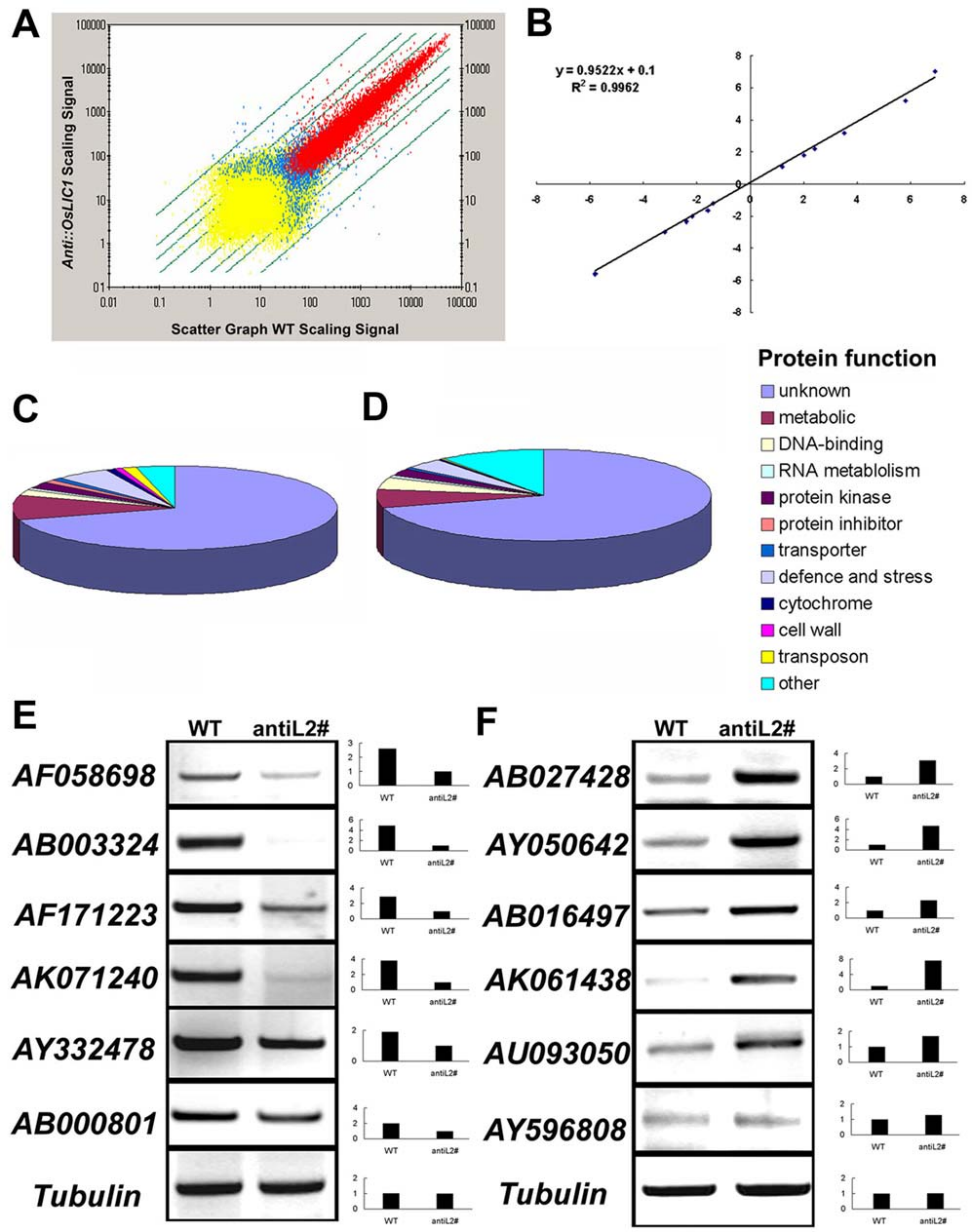
Global analysis of gene expression in antisense *OsLIC* transformants. (A) Scatter graph of signaling. Spots with absent, marginal or present detection signals are in yellow, blue or red color respectively. Only spots with a “Present” signal were used to determine the false-positive rate. (B) and (C) Predicated functions of the proteins encoded by up-regulated and down-regulated genes in collar of transgenic plants compared to those of wild-type plants on microarray analysis. RT-PCR analysis confirmed chip results. (E) Down-regulated genes in transgenic plants; (F) up-regulated genes in transgenic plants.

To further understand how OsLIC is involved in controlling rice plant architecture, we performed whole-genome expression profiling using the rice Affymetrix whole-genome microarray chip. The cRNAs from the developing collar in *AntiOsLIC* lines and the wild type were labeled as probes. The reliability of chip assay was confirmed by signaling scatter graph and RT-PCR (Figures 5A and 5B). All the RT-PCR primers were listed in Table S1. A total of 685 genes were up-regulated (at least 2-fold difference) and 490 genes were down-regulated (at least 2-fold difference) in collars of the antisense transgenic plants as compared with wild-type plants (NCBI online materials, GSE12067). The detected genes are mainly involved in cell-wall assembly and signaling transduction (Figure 5C and 5D). For example, genes involved in expansion (AF261274, AK059638, tigr: 9636.m03813), xylanase inhibitor protein (tigr: 9634.m02475, tigr: 9634.m02477) and pectate lyase (tigr: 9632.m00416) were greatly upregulated in the collar of the *AntiOsLIC* (NCBI online materials, GSE12067 and Figure 4B). This result suggested that the increased leaf angle in transgenic plants might be due to up-regulation of genes associated with cell-wall assembly. In addition, three genes upregulated in the collar of *AntiOsLIC* lines were previously known to be upregulated by BRs in rice [33]. These three genes respectively encode pyruvate decarboxylase (AK100678), H+-transporting ATPase (BI809899) and glutathione S-transferase (AF309376, AK062937, AK108376, AF309379, AF402799, AF309378, NM_193876, CR279062 and AF309382) (Figure 4B and Figure S2). Some genes associated with ethylene biosynthesis and signal transduction were reported to be significantly increased on activation of BR signal transduction [34]. Actually, putative 1-aminocyclopropane-1-carboxylate oxidase (AK102472), putative ethylene-forming enzyme (AK066303) and ethylene response factor ERF1 (AK108503) were expressed at higher levels at the level of transcription in transgenic plants (Figure S2). Those genes known to be up-regulated by BRs were also up-regulated in the antisense transgenic plants suggests that *OsLIC* may be involved in BRs response, probably through altering sterol abundance in transgenic plants.

### Expression of *OsLIC* can be induced by 24-epibrassinolide

**Figure 6:**
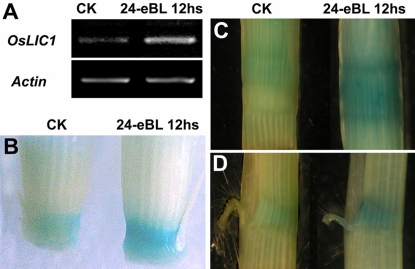
OsLIC is induced by 24-epibrassinolide at the transcriptional level. (A) mRNA level of *OsLIC* was significantly increased after treatment for 12 h with 1 µM 24-epibrassinolide. The GUS activity was enhanced in different tissues, including basal stem in tillering stage (B), nodes (C), and collar (D) after treatment.

To determine how *OsLIC* affects BR signaling in rice, we tested the expression pattern of *OsLIC* with exogenous BR treatment. We treated the basal region of wild-type culm at tillering stage for 12 h with 24-epibrassinolide, the most bioactive form of BRs, and found *OsLIC* expression significantly enhanced after the treatment (Figure 6A). In addition, we treated *OsLICpromoter::GUS* transgenic plants with 24-epibrassinolide for 12 h and found much stronger GUS activity after treatment than with mock treatment. The 24-epibrassinolide-induced expression pattern is consistent with the hypothesis that OsLIC is involved into the BR response in rice.

### *OsLIC* encodes a transcription activator

**Figure 7:**
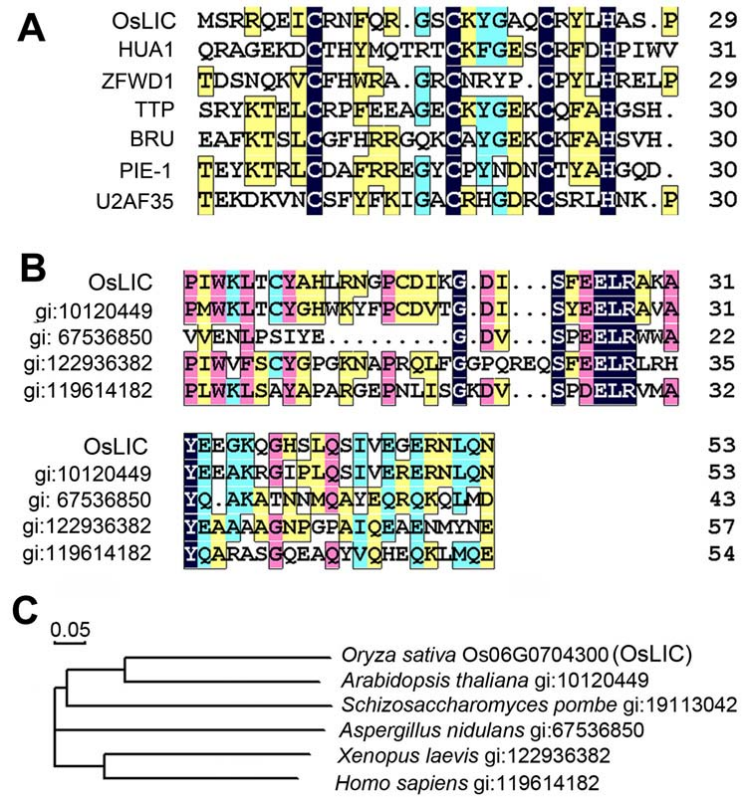
Annotation of *OsLIC*. (A) CCCH domain alignment of OsLIC with other CCCH-type zinc finger proteins demonstrated that OsLIC is a CCCH zinc finger protein. (B) Alignment of OsLIC with other proteins in its novel and conserved EELR domain. (C) Phylogenic tree of OsLIC and its orthologs in eukaryote organisms.

**Figure 8:**
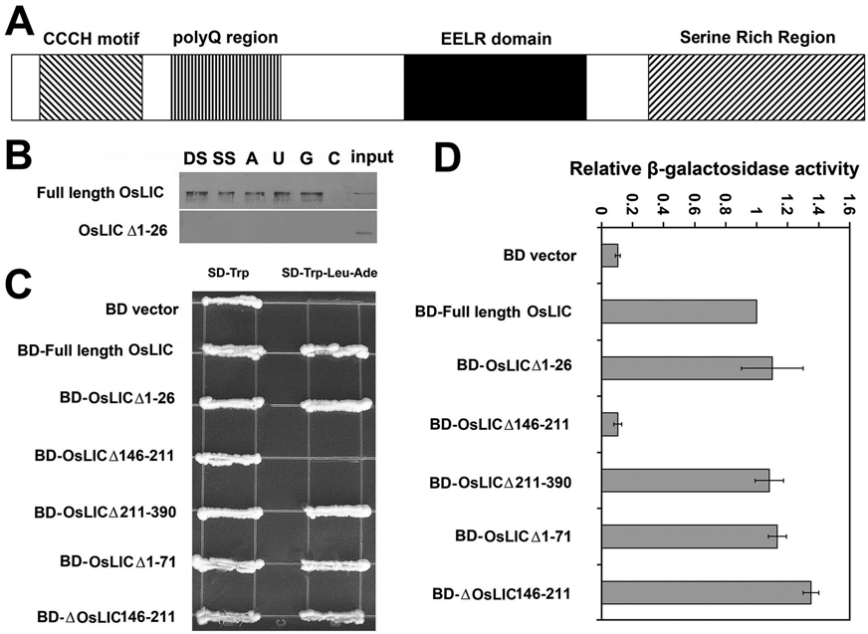
Biochemical character of OsLIC. (A) Schematization of *OsLIC* molecular structure. (B) Nucleic acid *in vitro* binding assay revealed that *OsLIC* binds with double-stranded DNA (DS) or single-stranded DNA (SS) and polyrA (A), polyrU (U), and polyrG (G) but not polyrC. (C) *OsLIC* displayed transcriptional activity in yeast, and the minimal activation domain is the conserved EELR domain (C, D).

**Figure 9:**
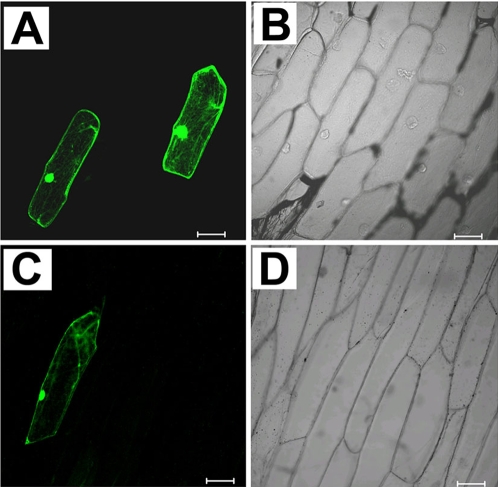
Intracellular localization of OsLIC. The binary vectors containing 35S::OsLIC-GFP or 35S::GFP were bombarded into onion epidermis cells to investigate subcellular localization of OsLIC protein. The lower left panel displays the signal from 35S::OsLIC-GFP. The upper left panel shows 35S::GFP signal alone. The excitation wavelength for GFP detection was 488 nm. The right panels are bright fields of left panels.

Bioinformatics analysis demonstrated that OsLIC protein contained a single C-x8-C-x5-C-x3-H (CCCH)-type zinc-binding motif at its N-terminus (Figure 7A). It also contained a novel EELR domain conserved in many eukaryote organisms (Figure 7B and 7C), as well as 4 tandem SSF motifs at its C-terminus (Figure 8A). Alignment assay of orthologs from *Arabidopsis* (gi: 10120449), *Schizosaccharomyces pombe* (gi: 191113042), *Xenopus laevis* (gi: 122936382) and *Homo sapiens* (gi: 119614182), showed them all to contain a single CCCH-type zinc finger motif in their N-terminus, which suggests that the putative proteins are highly conserved in eukaryotes. In addition, high conservation was observed among a 50 amino acid region flanking the core EELR motif (Figure 7B). Nucleic acid *in vitro* binding assay demonstrated that OsLIC binds to double-stranded DNA and single-stranded polyA, polyU and polyG but not polyC (Fig. 8B). Moreover, OsLIC with a truncated CCCH motif failed to bind any nucleic acid, which indicates that the CCCH motif is required for its nucleic acid binding activity (Figure 8B). Interestingly, OsLIC was shown to function as a transcription activator in yeast (Figure 8C and 8D). Analysis of a series of OsLIC deletions showed that the EELR domain was essential for the transcriptional activation. In addition, green fluorescent protein (GFP) fused with OsLIC showed expression in the nucleus and cytoplasm (Figure 9).

## Discussion

Higher plants display a variety of architectures that are defined by the degree of branching, internodes elongation and shoot determinacy [5]. Rice cultivars with desirable architectures are able to produce higher grain yields. Our findings demonstrate that OsLIC is a major regulator of rice architecture by functioning as a fine-tuning regulator of sterol homeostasis. Moreover, biochemical data suggest that OsLIC might be a direct transcription activator in controlling plant architecture.

### *OsLIC* is a major regulator of rice plant architecture

After the “Green Revolution” gene, OsGA20ox2, was isolated and characterized, many other essential genes critical to maintaining optimal plant architecture in rice have been characterized. These genes are mainly responsible for branching number and degree, dwarfism and leaf angle. *MOC1* functions to initiate auxiliary buds and to promote their outgrowth to control rice tillering [1]. Unlike the *moc1* mutant, the loss-of-function *OsTB1* mutant exhibits enhanced lateral branching in rice [35]. *OsTB1* encodes a putative transcription factor carrying a basic helix-loop-helix type of DNA-binding motif (named the TCP domain) and functions as a negative regulator of the outgrowth of tillering buds [35]. Tillering angle is also critical for dense planting because a wide tiller angle will increase leaf shade and decrease photosynthesis efficiency, whereas a narrow tiller angle is more efficient in plant architecture.

Optimal leaf angle is also necessary for dense planting. Rice BR biosynthesis and signaling mutants display an erect leaf phenotype, which is desirable for dense planting in rice grain production [10], [12], [13], [36]. However, many of these mutants also show a reduced grain-production phenotype. The weakest allele of OsBRI1 (*d61-7*) produces plants with higher biomass than wild-type plants under dense planting conditions but lower biomass under normal planting conditions [37]. Similarly, a rice BR-deficient mutant, *osdwarf4-1*, is associated with enhanced grain yield under conditions of dense planting because of its erect leaves, even without extra fertilizer [2]. Unlike the aforementioned mutants, the antisense *OsLIC* transgenic lines displayed multiple phenotypes, including increased leaf and tillering angle, semi-dwarfism, and decreased number of seeds in each panicle, which indicates that *OsLIC* plays critical roles in rice plant architecture. *OsLIC* is also involved in the regulation of rice panicle morphology. Antisense *OsLIC* transgenic lines show reduced number of spikelets and seeds in each panicle (Figure 1A and Table 1). The pattern of *OsLIC* expression, which is mainly in tissues containing lateral meristems and intercalary meristems, is consistent with the multiple defective phenotypes of the transgenic plants. Our results strongly suggest that *OsLIC* plays critical roles by influencing development of lateral and intercalary meristems, which contribute to regulating rice plant architecture.

### OsLIC is a negative regulator of the BR response

Antisense *OsLIC* transgenic plants displayed greatly increased leaf angle, a classical phenotype caused by overdose of BR or enhanced BR signaling in rice. Genetic analysis indicated that the mutation of *OsBRI1* (*d61-1*) [10] is epistatic to OsLIC (Figure 1H), which suggests that the increased leaf angle in *OsLIC* transformants might be due to activated BR synthesis. Sterol profile analysis demonstrated that shoots of the *OsLIC* transformants contain a higher level of both typical and atypical sterols, which further supports the above hypothesis. In addition, genome-wide expression analysis revealed that many genes identified as being BR induced showed higher expression levels at the transcriptional level. These genes include a number of cell-wall modifier genes (Figure 7). Recently, a few novel BR signaling components, besides the conserved components in both monocots and dicotyls, were identified and characterized [10], [29], [38]–[40]. Among them, three MADS-box genes with short vegetative phase (*OsMADS22*, *OsMADS47* and *OsMADS55*) were shown to be involved in the BR response in rice [39], [41]. Casein kinases are critical in cell division and differentiation across species. Casein kinase I is involved in the BR signaling pathway in rice [42]. Unlike the *OsBRI1* mutants such as *dm*-type or *d6*-type dwarfism, the dwarfism of the *OsLIC* antisense line was a *dn*-type dwarf [10], which is caused by shortening of all internodes, not just particular ones (Figure 1F). Because stem elongation is notably reduced with BR oversensitivity in rice [39], the semi-dwarfism phenotype in *AntiOsLIC* transgenic plants might be caused by the enhanced BR response. In agreement with this point, our previous results indicated that ectopic expression of Arabidopsis *BAK1* driven by a maize ubiquitin promoter in rice also caused the dwarfism because of enhanced BR signaling [29]. The overall levels of intermediates that are important for sterol biosynthesis in plants were greatly increased in *AntiOsLIC* transgenic plants. Thus, the multiple defective phenotypes of OsLIC transformants are due to activated BR synthesis and OsLIC function as a negative regulator of the BR response in rice. However, the detailed mechanism of OsLIC regulating sterol homeostasis still needs further investigation. Such investigation could involve more genetic crosses of *AntiOsLIC* transgenic plants with identified rice sterol biosynthesis mutants. In addition, addressing this question will be helpful in identifying the genes OsLIC directly targets as a transcription factor.

### *OsLIC* encodes a novel transcription activator as a CCCH zinc finger protein

OsLIC is a member of the CCCH zinc finger protein family but has a single CCCH motif, which is distinct from other CCCH-type zinc finger protein members such as TTP/TIS11/NUP475, PIE-1, POS-1 in animals, and HUA and FES1 in Arabidopsis. Our data suggest that OsLIC can bind to polyrA, polyrU and polyrG under intermediate salt concentrations (Figure 8). OsLIC can also bind both double-stranded and single-stranded DNA via *in vitro* nuclear binding assay. However, OsLIC protein contains another conserved domain with core amino acid sequence EELR in eukaryotes. The EELR domain is associated with transcriptional activation (Figure 8C and D). The presence of this domain indicates that OsLIC can function as a novel transcriptional activator.

In summary, our findings indicate that OsLIC is required for fine-tuning the modulation of sterol abundance and functions as a regulator to maintain the optional morphology in rice, which is essential for high-yield grains. OsLIC with a CCCH zinc finger motif is the first reported negative regulator mediating rice architecture. It may provide a novel strategy to improve rice plant architecture for higher yields in the near future.

## Materials and Methods

### Construction of transgenic plants

To generate an antisense expression vector, *OsLIC* gene was constructed to be driven by a ubiquitin promoter in the binary vector pUN1301[43] in the antisense direction, which created *pAntiOsLIC*. It was transferred into *Agrobacterium tumefaciens* EHA105. Rice embryonic calli induced from germinated seeds were used for *Agrobacterium*-mediated rice plant transformation as described previously [44]. Transgenic plants were selected in half-strength Marushige and Skoog (MS) medium containing 75 mg L^−1^ hygromycin (Sigma).

### Histochemical GUS activity Assay

GUS staining was performed as described [45]. Tissues of transgenic seedlings harboring *OsLIC:::GUS* were incubated in a solution containing 50 mM Na_3_PO_4_ buffer (pH 7.0), 5 mM K_3_ Fe (CN)_6_, 5 mM K_4_ Fe (CN)_6_, 0.1% Triton X-100, and 1 mM X-Gluc at 37°C for 4 hr. All samples were vacuum infiltrated for 5 min before the staining process.

### Affymetrix GeneChip Analysis

The developing collar from the *OsLIC* antisense transgenic plants and the wild type was harvested at the heading stage. The position of the collar was about 1 cm above the last developed collar. Total RNA was isolated by use of TRIzol reagent (Invitrogen) and purified by use of Qiagen RNeasy columns (QIAGEN). For Affymetrix GeneChip analysis, 8 µg of total RNA was used for making biotin-labeled cRNA target. All processes for cDNA and cRNA synthesis, cRNA fragmentation, hybridization, washing and staining, and scanning, were conducted according to the GeneChip Standard Protocol (Eukaryotic Target Preparation, Affymetrix). Information on GeneChip® Rice Genome Array (MAS 5.0) was accessed from the Affymetrix website: http://www.affymetrix.com/products/arrays/specific/rice.affx. Affymetrix GeneChip Operating Software (GCOS) was used for data collection and normalization. The overall intensity of all probe sets of each array was scaled to 500 to ensure equal hybridization intensity; each probe set was assigned “P”, “A” or “M” and a p value from the algorithm in GCOS. To identify genes with differential expression pattern in the *OsLIC* antisense transgenic lines and wild-type plants, a log2-transformed signal ratio of each gene was calculated with use of the GCOS baseline tool, and log2 (ratio) ≥1 (2-fold change) was used as a cut-off.

### Genomic DNA isolation and DNA Gel Blot Analysis

Genomic DNA isolation and DNA gel blot analysis were performed as described [43]. Genomic DNA of 30 µg was digested by use of *Eco*RI, *Bam*HI and *Hin*dIII. The fragments were separated on 0.7% agarose gel by electrophoresis. The separated DNA fragments were transferred and cross-linked onto a nylon membrane (Hybrid N^+^; Amersham, Buckinghamshire, UK), as described [46]. The probe was labeled with ^32^P-dCTP (China Isotope, Beijing) and synthesized by PCR with the following primers 5′- AAGTACGGAGCGCAGTGCAG -3′ and 5′-T TCCATGATTCCATCCCT T-3′. To detect the insertion events, 30 µg genomic DNA from wild-type and transgenic plants was digested by *Xba*I. The probe was synthesized with use of primers 5′-GCATGATACGTCCTGTAGAAACCC-3′ and 5′-CAAAGCCAGTAAAGTAGAACGGT-3′ after hybridization with *UidA* gene.

### *In situ* hybridization

Tissues were fixed in 4% (w/v) paraformaldehyde and 0.25% glutaraldehyde in 0.1 M sodium phosphate buffer; samples were vacuum infiltrated for 30 min and then stored overnight at 4°C. The dehydrated samples after a graded ethanol series were embedded in Paraplast Plus (Oxford Labware, St. Louis, MO). A fragment of 232 bp was amplified from the second exon of *OsLIC* with the primers for *Bam*HI and *Hin*dIII sites (underlined) 5′-GGATCCGCAAGTACGGAGCGCAGTG-3′ and 5′-AAGCTTTTCGCAGGACCAGGAGCA-3′, respectively, then was subcloned into pGEM-T-easy vector (Promega) and used as a template for RNA probe synthesis. *In situ* hybridization with digoxigenin-labeled sense or antisense RNA of *OsLIC* was conducted as previously described [47].

### Protein Immunoblot Analysis

The full ORF of OsLIC was fused with GST in a pGEX-4T-1 vector. The OsLIC fusion protein was expressed in *Escherichia coli* and purified with use of Sepharose-4B beads according to the protocols (Amersham). Purified protein of 2 mg was injected into rabbit to raise an anti-OsLIC serum. For protein gel blot analysis, the young stems from transgenic plants and wild-type plants at the tillering stage were harvested, weighed and homogenized in SDS loading buffer (0.2 M Tris-HCl, pH 6.8, 0.5 M DTT, 4% SDS, and 25% glycerol). After being boiled for 5 min and centrifuged, the proteins were separated on 12% Tris-Tricine gels and blotted onto Nitrobind (Micron Separations, Westborough, MA) by use of a Bio-Rad Transblot SD wet electroblotting apparatus (Hercules, CA). Blots were treated with the rabbit anti- OsLIC serum (1∶200). The immune complex was detected by alkaline phosphotase-conjugated secondary antibodies and nitroblue tetrazolumy 5-bromo-4-chloro-3-indolyl phosphate (Promega).

### Phytosterol abundance analysis

The lyophilized rice shoots (10–12 g) and roots (5–7 g) were homogenized with use of liquid nitrogen, extracted with methanol, and then chloroform. After adding deuterium-labeled (^2^H_7_-labeled) cholesterol (1 µg) as an internal standard, the chloroform-soluble extracts were dried and saponified with 5% ethanolic KOH for 2 hr at 70°C. The unsaponified lipids were extracted with use of n-hexane (20 ml×2) and dried in a vacuum. The dried residues were loaded on a Sep-Pal cartridge and eluted with n-hexane-ethyl acetate (3∶2, 8 ml). The obtained fractions were acetylated with pyridine-acetate anhydride (2∶1, 1 ml) for 18 hr at room temperature. The acetylated phytosterols were extracted with use of n-hexane (2 ml×3), and analyzed by GC-MS under the following conditions: Hewlett-Packard GC-MS (6890-5973); capillary column, HP-5 (30 m×0.25 mm i.d., 0.25 µm film thickness, J&W Scientific); column temperature 150°C for 4 min, thermal gradient 10°Cmin^−1^ to 280°C, and then 280°C; carrier gas helium, with flow rate, 1 ml/min. The levels of endogenous sterols were determined on the basis of calibration curves constructed from the ratios of the M+ peak area of ^2^H_7_-labeled cholesterol added as an internal standard.

### Trans-activation Activity Assay

The full-length coding region and various deletion mutants of *OsLIC* were amplified by use of a polymerase with high proofreading activity (*Pfx*, Invitrogen) and appropriate primers as follows:

OsLIC (1)-F: 5′-CGGAATTCATGAGTCGGCGGCAGGAGATTTGCC-3′,
OsLIC (26)-F: 5′-CGGAATTCGCCTCCCCTCACCAGCAA3′,
OsLIC (146)-F: 5′-CGGAATTCTCCTATTTGGAAGCTTACTTGTTA-3′
OsLIC (211)-R: 5′-CGGGATCCCCCACATTCATGTTCCAGG-3′
OsLIC (390)-R: 5′-CGGGATCCTTAAAACACATGGCTAACGTGACTC-3′

The numbers in the brackets indicate the corresponding amino acid sites for respective primers and underlined nucleotides correspond to *Eco*RI and *Bam*HI sites. The truncated EELR domain was constructed by RT-PCR. Full-length cDNA of *OsLIC* was inserted into the pGEM-Teasy vector and then reverse amplified with the following primers: EELRT-F 5′-GAA GAT CTT ATG AGG AAG GAA AGC AAG GGC ATT-3′ and EELRT-R 5′-GAAGATCTAGCCTCGTTCTTAAAATCCTCAGAA-3′; the underlined nucleotides correspond to the *Bgl*II site. The PCR products were digested by *Bgl*II and self-ligated to transform into *E. coli* DH5α competent cells. After the positive clones were identified, the truncated EELR domain construct was amplified with primers for *OsLIC* (1)-F and *OsLIC* (390)-R described above. Inserts were fused in-frame to the sequences encoding the GAL4 DNA binding domain by cloning them to pGBKT7. All the inserts of the recombinant plasmids were sequenced to confirm the veracity during PCR amplification. All the constructs with pGBKT7 blank vector were transformed directly into *Saccharomyces cerevisiae* AH109 by yeast LiAc-mediated transformations according to the protocols (Clontech, Palo Alto, CA). Yeast transformants were screened by dropout SD-Trp. SD/-Trp-His-Ade screening combined with β-galactosidase filter assays using X-gal was performed to test autonomous activation.

### *In Vitro* Nucleic Binding Assay

The full-length and truncated CCCH-domain *OsLIC* were cloned into pGEX-4T-1 vector. The proteins were purified with Sepharose-4B beads according to the protocols (Amersham). Purified protein of 0.5 µg was incubated with 20 µL of poly rA, poly rG, poly rC, and poly rU attached to agarose beads and double-stranded and single-stranded calf thymus DNA attached to cellulose beads (Sigma) in 500 µL of RHPA binding buffer (10 mM Tris, pH 7.4, 2.5 mM MgCl_2_, 0.5% Triton X-100, and NaCl at various concentrations) with 1 mg/mL heparin. After incubation at 4°C for 10 min, the beads were washed five times in RHPA buffer and then boiled in SDS loading buffer. The proteins were separated by SDS-PAGE and underwent immunoblot analysis.

### Subcellular Localization

The full-length *OsLIC* coding region without a stop codon was subcloned to the N terminus of green fluorescent protein (GFP) in the pBI221 vector. The *OsLIC::GFP* fusion construct vector and pBI221 vector were used to bombard onion epidermis cells with use of the Bio-Rad biolistic system (Hercules, CA). After culture for two days, the epidermis was visualized with use of a laser scanning confocal microscope (LSM 510, Zeiss, Oberkochen, Germany). The excitation wavelength for GFP detection was 488 nm[29].

## Supporting Information

Table S1 PCR primers were used for RT-PCR
(0.05 MB DOC)

Figure S1 Molecular characterization of transgenic plants. (A). Southern blot analysis of three independent OsLIC transgenic lines containing a single-copy insertion in different insertion sites. (B). Immunoblot assay of endogenous OsLIC expression inhibited in all three transgenic lines to a different extent.
(2.14 MB TIF)

Figure S2 Semi-quantitative RT-PCR identified the genes involved in ethylene biosynthesis and signal transduction pathway.
(0.46 MB TIF)
