# Supplementary material for: OsLIC, a Novel CCCH-Type Zinc Finger Protein with Transcription Activation, Mediates Rice Architecture via Brassinosteroids Signaling
Source: PLoS One. 2008 Oct 27;3(10):e3521. doi: 10.1371/journal.pone.0003521 (PMC2567845; doi:10.1371/journal.pone.0003521)
Supplement: Table S1 — PCR primers were used for RT-PCR (0.05 MB DOC) [file pone.0003521.s001.doc]

**Supplemental Table 1: Primers were used for RT-PCR**

| **Gene_ID** | **Primers for Reverse transcriptional PCR** |
| --- | --- |
| AB000801 | Sense primer: 5’ GCCTGCGTGCTCTTCAT 3’  Antisense primer: 5’ CCAACCAAACCACCCATACA3’ |
| AB003324 | Sense primer: 5’ CCACTGGCTACAAATGAA 3’  Antisense primer: 5’ GGGAGGCTCACTGGAAA 3’ |
| AB016497 | Sense primer: 5’ GGCGACTTCTCCACCCTAC 3’  Antisense primer: 5’ GCCGCCGTTGATGATGTT 3’ |
| AB027428 | Sense primer: 5’ TAACACTGGCATTGGTCCTT 3’  Antisense primer: 5’ TGCCGACCGTGATGTTAGCG 3’ |
| AF058698 | Sense primer: 5’ TAAGGCAAAGATTGAGACC 3’  Antisense primer: 5’ GGTCCCACTGCCCTACTT 3’ |
| AF171223 | Sense primer: 5’ CTTGGCGATGGTTATGTC 3’  Antisense primer: 5’ CATAGTCCCATCCATAGTTT 3’ |
| AK061438 | Sense primer: 5’TGACTCGCCCTTTGATTT 3’  Antisense primer: 5’ ATGTTGGCAGCCGATGTG 3’ |
| AK071240 | Sense primer: 5’ TCAAGTCCTCCGACATCAAG 3’  Antisense primer: 5’ CACATCGCATACGGCATACA 3’ |
| AU093050 | Sense primer: 5’ AAGAAGAGGGTGGAACGG 3’  Antisense primer: 5’ CACAGCAGCACAGCATTT 3’ |
| AYO50642 | Sense primer: 5’ ATGGACCGCCTTCTGTGG 3’  Antisense primer: 5’ GAGTGCCAACCTCTTCCA 3’ |
| AY332478 | Sense primer: 5’ GCTGGCGAAAGGATAGAG 3’  Antisense primer: 5’ AATGCTTTCCTCGTCTCG 3’ |
| AY596808 | Sense primer: 5’ TTTTCAAACAACGCAAAGCAGTAG 3’  Antisense primer: 5’ CAGCACCCAATCATCCAACCT 3’ |
| AF261274 | Sense primer：5' AGCCCATCTTCAAGGACG 3'  Antisense primer：5' ACCTCAATCCCACGCAAA 3 |
| AF309376 | Sense primer: 5' GTGATGCCCTCTGACCCCTA 3'  Antisense primer: 5' GCAGCAGCATTTCTCCTTGTTA 3' |
| AK059638 | Sense primer: 5' TACAAGGGAGGCAAGGGGT 3'  Antisense primer: 5' CCTCTTCTGACACCGCCTCT 3' |
| AK062612 | Sense primer: 5' GTGGTAGTGACCCTGCTGAC 3'  Antisense primer: 5' AAGTAGGTCCTTGTGTCCGATA 3' |
| AK062937 | Sense primer: 5' CAGTGCTTCATCGTCCCCA 3'  Antisense primer: 5' TCACCTTCTTCACGCTCGG 3' |
| AK066303 | Sense primer: 5' ATGGTGAGGCGATTCCGA 3'  Antisense primer : 5' GCGAAATGCCCATCACCT 3' |
| AK102472 | Sense primer: 5' GACTGGGGCTTCTTCTGGGT 3'  Antisense primer : 5' TGGTGCCCTGGTAGTAGTCC 3' |
| BI809899 | Sense primer: 5' CGGCTTCACCATCCAGTA 3'  Antisense primer: 5' AGAGACAACAGGAGGAGAGGA 3' |
| CK738253 | Sense primer: 5' TAATAATGGAGCTCGCAAGC 3'  Antisense primer : 5' TGGCAGTGCTTGATGTCG 3' |
| NM196827 | Sense primer: 5' ACGGCATCTCGCTCTACTCG 3'  Antisense primer: 5' GGTTGCCCTGGCTGTTGAT 3' |
| U07339 | Sense primer: 5' GGCGACTCGTGGTTCAA 3'  Antisense primer : 5' TGCCGTCAGAGTTGTGGA 3' |
