# Supplementary figures and images for: OsLIC, a Novel CCCH-Type Zinc Finger Protein with Transcription Activation, Mediates Rice Architecture via Brassinosteroids Signaling
Source: PLoS One. 2008 Oct 27;3(10):e3521. doi: 10.1371/journal.pone.0003521 (PMC2567845; doi:10.1371/journal.pone.0003521)

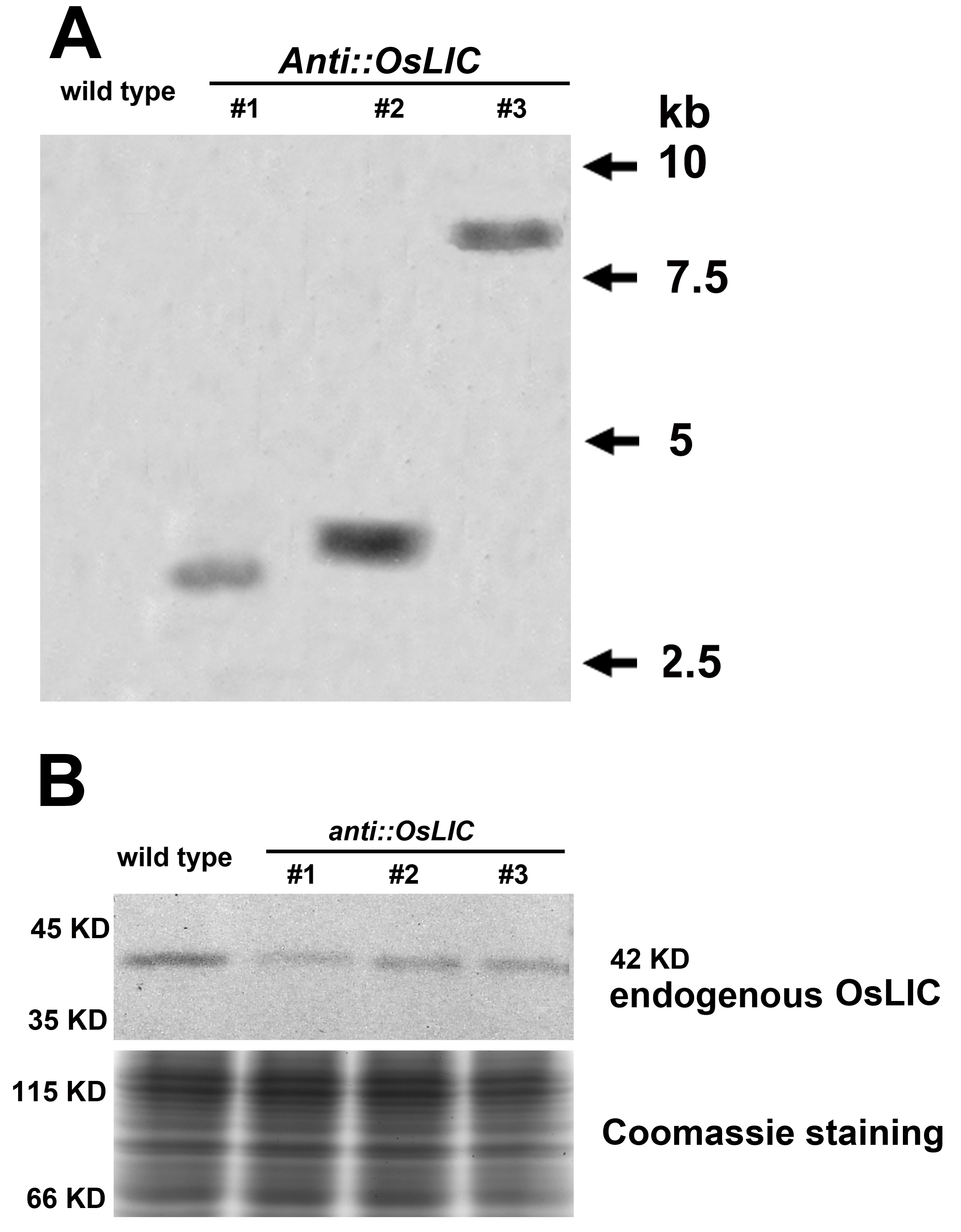

Supplement: Figure S1 — Molecular characterization of transgenic plants. (A). Southern blot analysis of three independent OsLIC transgenic lines containing a single-copy insertion in different insertion sites. (B). Immunoblot assay of endogenous OsLIC expression inhibited in all three transgenic lines to a different extent. (2.14 MB TIF) [file pone.0003521.s002.tif]

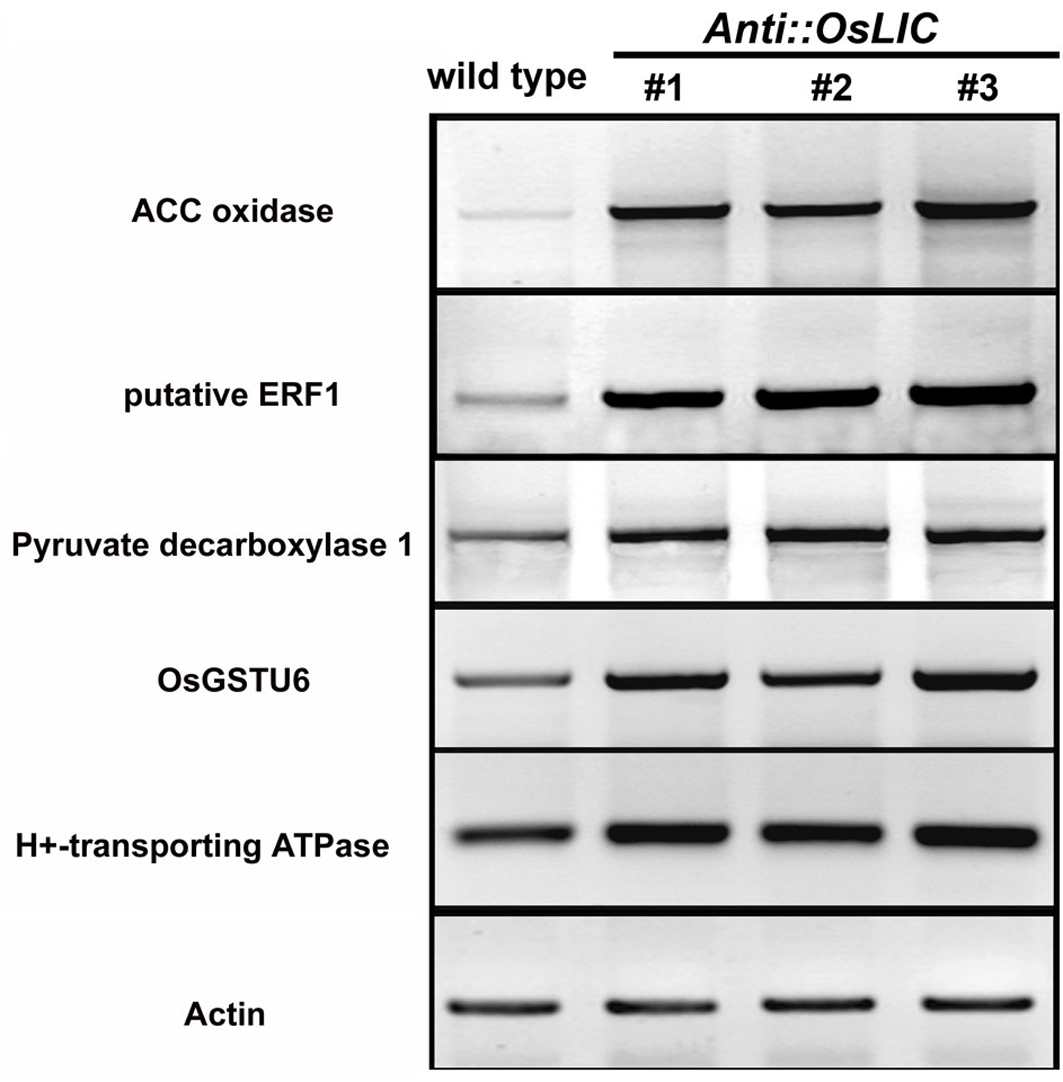

Supplement: Figure S2 — Semi-quantitative RT-PCR identified the genes involved in ethylene biosynthesis and signal transduction pathway. (0.46 MB TIF) [file pone.0003521.s003.tif]
